# Super giant basal cell carcinoma in the setting of neglect: A case report

**DOI:** 10.1016/j.jdcr.2026.05.015

**Published:** 2026-05-12

**Authors:** Henry Lim, Alyssa Forsyth, Marshall Hall, Christian Scheufele, Dustin V. Wilkes

**Affiliations:** a Department of Dermatology, Medical City Fort Worth, Fort Worth, Texas; b University of North Texas Health Science Center, Texas College of Osteopathic Medicine, Fort Worth, Texas

**Keywords:** hedgehog pathway inhibitor, locally advanced basal cell carcinoma, sonidegib, super giant basal cell carcinoma

## Introduction

Super-giant basal cell carcinomas(SGBCCs) are rare, defined as ≥20 cm, with very few reported in the literature.^1^ These extensive tumors pose a therapeutic challenge, requiring multidisciplinary team efforts for both management and prevention strategies. Hedgehog pathway inhibitors (HHI) are effective therapies for locally advanced BCC.^2^

Here, we present a case of SGBCC undergoing successful treatment with HHI. This case illuminates the multifactorial drivers for tumor progression. To our knowledge, this is one of the largest reported SGBCC being treated successfully with HHI monotherapy to date.^1^ We also discuss opportunities for earlier intervention and prevention to reduce the likelihood of progression to advanced, debilitating disease.

## Case presentation

**Fig 1:**
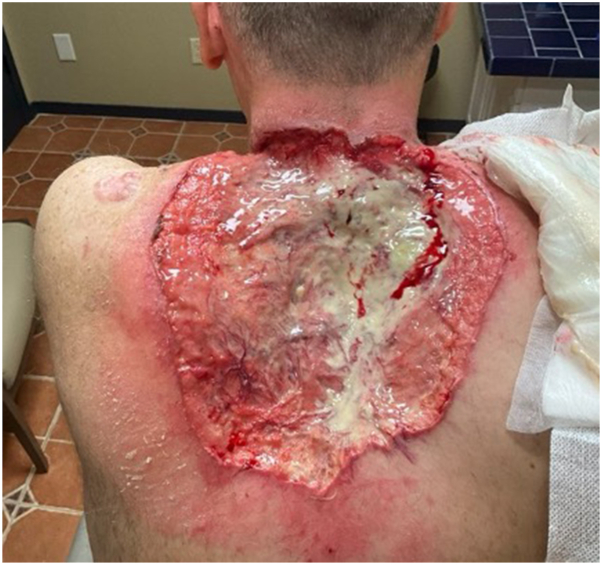
Clinical presentation at month 0. Initial presentation demonstrating a large, ulcerated, and bleeding plaque with rolled borders exposing underlying muscle. A separate pigmented, waxy plaque with telangiectasia on the left shoulder is also noted, suspicious for basal cell carcinoma.

A 58-year-old male presented with a 25 × 25 cm nonhealing ulcerated plaque with rolled borders on the upper back (Fig 1). He was an ex-competitive bodybuilder with a history of extensive tanning bed use and numerous nonmelanoma skin cancers (NMSCs). This lesion was biopsied 11 years prior and found to be a nodular basal cell carcinoma; however, the patient had neglected treatment due to surgical fatigue from his numerous other NMSC treatments. The patient ultimately perceived his BCCs as low risk and chose to discontinue formal care.

Prior to presentation, at-home treatment included solasodine rhamnosyl glycosides without improvement. He ultimately sought care due to progressive growth and pain requiring daily ibuprofen, prompted by concern of his spouse.

Physical examination revealed a large pink ulcerated plaque with rolled borders engulfing the majority of the upper back. Other suspicious waxy plaques with telangiectasia were also noted scattered on the torso. He appeared nontoxic, afebrile, without palmoplantar pits, milia, or skeletal abnormalities. His lymph node examination was unremarkable, and he denied a history of odontogenic cysts. Review of systems was negative for unintentional weight loss, weakness, or fatigue.

His baseline lab values, including complete blood count, comprehensive metabolic panel, and creatine kinase, were within normal limits. The patient declined staging imaging due to financial constraints and declined genetic testing.

**Fig 2:**
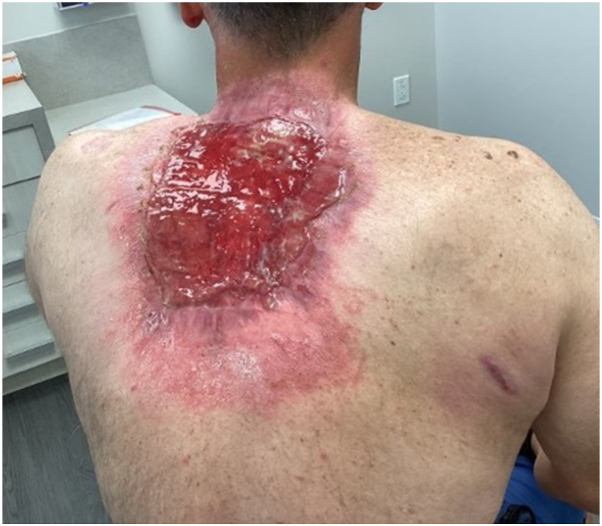
Clinical presentation at month 4. Partial clinical response following initiation of sonidegib, with decreased ulceration and early re-epithelialization.

**Fig 3:**
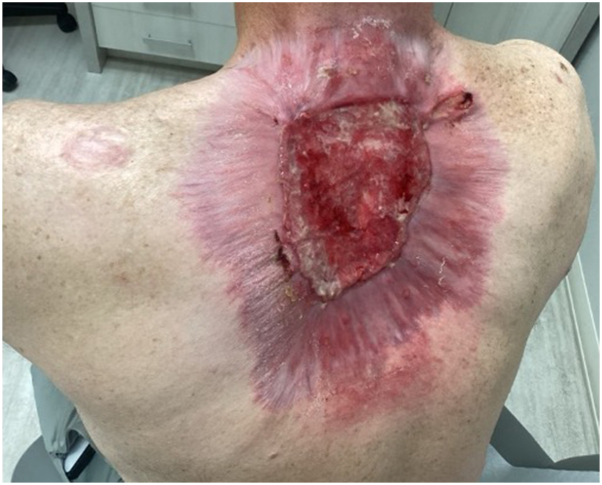
Clinical presentation at month 7. Continued re-epithelialization of the majority of the plaque, evidenced by scar tissue arranged in parallel fibers.

He was initiated on sonidegib 25 mg daily with L-carnitine 1000 units supplementation. By month 7 of therapy, the patient had a visibly substantial improvement in tumor burden and quality of life (Figs 2 and 3). Repeat labs revealed no concerning abnormalities. Patient denied adverse effects, including muscle spasm, alopecia, or dysgeusia.

## Discussion

There are no standardized guidelines for the management of large, locally advanced skin cancers.^1^ In the setting of treatment fatigue, as in this case, modalities that minimize operative burden may assume priority in shared decision-making. Patients with keratinocyte carcinomas may experience cumulative treatment fatigue due to repeated procedures, chronic surveillance, and therapy-related adverse effects. This can decrease adherence and reduce willingness to pursue continued care.^2^

Hedgehog pathway inhibitors (HHIs) are indicated in the management of giant or multiple BCCs that cannot practically be treated with surgery or radiation alone.^2^^,^^3^ Vismodegib and sonidegib are FDA-approved oral smoothened inhibitors approved for locally advanced BCC that has recurred or is unsuitable for surgery or radiation.^2^ Vismodegib is FDA-approved for metastatic BCC as well.^2^ HHIs may be able to serve as neoadjuvant therapies to decrease tumor burden before surgery or radiation, although they are not yet approved for this indication, and further studies must be conducted.^2^^,^^3^ Some disadvantages of HHI therapy are the adverse effect profile and the risk of creating resistance.^2^^,^^3^ The most commonly observed adverse events were muscle spasms, alopecia, dysgeusia, fatigue, weight loss, nausea, decreased appetite, and diarrhea.^2^

Adverse events from HHI therapy may be managed through strategies such as treatment interruptions, dose reductions, and adjunctive therapies like L-carnitine.^4^ Failure to manage these side effects often leads patients to discontinue therapy prematurely, reducing treatment efficacy.^2^ In this case, L-carnitine supplementation may have contributed to the absence of muscle spasms. The patient also did not experience alopecia or dysgeusia. While a 7-month treatment duration may have been insufficient for these to become intolerable, alopecia and dysgeusia have reported median times to onset of 5.5 and 3.7 months, respectively.^2^

Germline genetic testing for mutations in patched 1 or suppressor of fused genes is warranted in patients with giant or multiple BCCs, especially in this case of an SGBCC.^5^^,^^6^ Although the patient declined genetic evaluation, the clinical response to HHI suggests that the BCC pathogenesis is mediated by patched 1rather than suppressor of fused, as suppressor of fused mutations would not be expected to respond to HHI.^5^ However, whether the patient has basal cell nevus syndrome remains unclear.

Strategizing intervention for patients with large or numerous skin cancers is important following a period of dermatologic neglect. While BCC usually has a favorable prognosis, advanced cases, such as SGBCC, have a higher risk of metastasis and can be highly aggressive.^7^ For these patients, the 5-year disease-specific survival rate is 79% for locally advanced BCC and 30% for metastatic disease.^7^ Many of these advanced cases could have easily been treated at earlier stages with curative excision or other available means.^6^ Therefore, heightened awareness among the public and healthcare professionals is crucial for timely intervention.^6^

This case demonstrates the importance of early intervention and the effectiveness of sonidegib in SGBCC. Preventing neglect requires public health measures and patient education.^8^ During shared decision-making, emphasis on the low mortality of NMSCs may inadvertently encourage patients to opt for more conservative strategies or neglect treatment altogether. Public health messaging should better convey the potential severity of untreated NMSCs to help patients properly prioritize care.^8^

The development of giant skin cancers reflects a constellation of patient-related factors that extend beyond simple negligence, as illustrated by this case.^9^ The patient prioritized treatment of other skin cancers, perceived the basal cell carcinoma as low-risk, and attempted alternative home remedies. Two common patient patterns that contribute to giant skin cancer presentations are those with delayed presentation to medical care and those with prior attempts at management using inappropriate strategies.^9^ This patient represents both. The progression of these tumors can be driven by self-neglect, denial, avoidance behavior, misinterpretation of symptoms, and fear of treatment.^9^^,^^10^ Factors such as surgical fatigue and tumor size guide the treatment plan.

### Declaration of generative AI and AI-assisted technologies in the writing process

No AI was used in the drafting, analysis, or revision of this manuscript.

## Conflicts of interest

None disclosed.
